# Temperature, by Controlling Growth Rate, Regulates CRISPR-Cas Activity in Pseudomonas aeruginosa

**DOI:** 10.1128/mBio.02184-18

**Published:** 2018-11-13

**Authors:** Nina Molin Høyland-Kroghsbo, Katrina Arcelia Muñoz, Bonnie L. Bassler

**Affiliations:** a Department of Molecular Biology, Princeton University, Princeton, New Jersey, USA; b Howard Hughes Medical Institute, Chevy Chase, Maryland, USA; University of Würzburg; NCI-NIH; Rockefeller University

**Keywords:** CRISPR, phage, *Pseudomonas*, quorum sensing, growth rate

## Abstract

Clustered regularly interspaced short palindromic repeat (CRISPR)-associated (CRISPR-Cas) systems are adaptive defense systems that protect bacteria and archaea from invading genetic elements. In Pseudomonas aeruginosa, quorum sensing (QS) induces the CRISPR-Cas defense system at high cell density when the risk of bacteriophage infection is high. Here, we show that another cue, temperature, modulates P. aeruginosa CRISPR-Cas. Increased CRISPR adaptation occurs at environmental (i.e., low) temperatures compared to that at body (i.e., high) temperature. This increase is a consequence of the accumulation of CRISPR-Cas complexes, coupled with reduced P. aeruginosa growth rate at the lower temperature, the latter of which provides additional time prior to cell division for CRISPR-Cas to patrol the cell and successfully eliminate and/or acquire immunity to foreign DNA. Analyses of a QS mutant and synthetic QS compounds show that the QS and temperature cues act synergistically. The diversity and level of phage encountered by P. aeruginosa in the environment exceed that in the human body, presumably warranting increased reliance on CRISPR-Cas at environmental temperatures.

## Abstract (precis)

P. aeruginosa is a soil dwelling bacterium and a plant pathogen, and it also causes life-threatening infections in humans. Thus, P. aeruginosa thrives in diverse environments and over a broad range of temperatures. Some P. aeruginosa strains rely on the CRISPR-Cas adaptive immune system as a phage defense mechanism. Our discovery that low temperatures increase CRISPR adaptation suggests that the rarely occurring but crucial naive adaptation events may take place predominantly under conditions of slow growth, e.g., during the bacterium’s soil dwelling existence and during slow growth in biofilms.

## INTRODUCTION

Bacteria have evolved defense systems to fend off bacteriophage viruses that prey upon them. One of these systems, the clustered regularly interspaced short palindromic repeat (CRISPR)-associated (CRISPR-Cas) system, provides acquired and heritable sequence-specific immunity against previously encountered viruses and plasmids (1, 2). CRISPR-Cas systems generally rely on three main activities, adaptation, expression, and interference (3). Upon infection with a foreign genetic element, the CRISPR-Cas machinery can incorporate a short piece of foreign DNA into the genomic CRISPR array, which contains the genetic memory of prior infecting elements. The CRISPR array thereby expands in size by gaining an additional repeat and a spacer derived from the foreign DNA. This process is known as adaptation (1, 4, 5). Next, during the expression stage, the CRISPR array is transcribed into pre-CRISPR RNAs (pre-crRNAs) that are processed into mature crRNAs, each complementary to a particular foreign DNA sequence. crRNAs guide Cas protein complexes to target complementary incoming foreign DNA, denoted protospacers (6). Most CRISPR-Cas systems require the presence of a short protospacer adjacent motif (PAM) to correctly identify the target DNA and distinguish it from self (7, 8). The final stage is interference, when Cas immune surveillance complexes, guided by mature crRNAs, cleave, and thereby eliminate, complementary foreign genetic material (9).

Naive CRISPR adaptation to a foreign genetic element that a bacterium has not previously encountered is rare in type I CRISPR-Cas systems (10). However, when a crRNA has partial complementarity to a protospacer sequence, the frequency of new spacer acquisition is enhanced more than 500-fold (11). This process is known as primed adaptation (5). Primed adaptation is crucial for type I CRISPR-Cas systems to robustly fight off contemporary threats, notably when the foreign element mutates, which would otherwise allow escape from CRISPR targeting (10). In this same vein, CRISPR arrays possessing multiple spacers targeting the same phage reduce the chances that a phage can acquire mutations that enable it to escape detection (12). This arrangement, moreover, allows a larger proportion of CRISPR-Cas complexes to be loaded with crRNAs that target a particular infecting phage, providing a more favorable CRISPR-Cas complex-to-phage target ratio, again increasing the success of the defense system.

Naive adaptation requires cleavage of the newly infecting DNA, the incorporation of a short fragment as a new spacer in the CRISPR array, transcription and processing of the new crRNA, and formation of a crRNA-Cas complex to scan the genomic and foreign DNA to pinpoint the foreign DNA and target it for cleavage. These steps take time. During that time, the phage is executing its parasitic program, either lysogenizing the host, in which case adaptation would lead to host suicide, or generating numerous copies of the phage genome as the phage prepares to lyse the host cell. With respect to the phage lysis program, it could be difficult for the CRISPR-Cas machinery to keep pace (degrading phage genomes as new phage genomes are produced), possibly a contributing feature underpinning why naive adaptation is so infrequent. Indeed, in line with this argument, defective phage particles capable of infecting but not killing host bacteria have been shown to increase adaptation frequency (13). In this case, a defective phage injects its DNA, which can serve as a substrate for naive adaptation, in a setting in which the race between host killing and CRISPR-Cas success does not occur.

Bacteria incorporate cues, such as nutrient availability, into the regulation of CRISPR-Cas (14–17). We previously discovered that the cue for cell population density is integrated into CRISPR-Cas regulation. Specifically, cell-cell communication, i.e., quorum sensing (QS), activates type I-F CRISPR-*cas* expression, CRISPR-Cas activity, and CRISPR adaptation in P. aeruginosa UCBPP-PA14 (here denoted PA14), enabling CRISPR-Cas function to increase in step with increasing bacterial cell density (18). This mechanism ensures maximum CRISPR-Cas activity when bacterial populations are at high cell density and at highest risk for phage infection and spread. Likewise, Patterson et al. showed that in Serratia sp. strain ATCC 39006, the QS autoinducer synthase SmaI is required for expression and activity of type I-E, I-F, and III-A CRISPR-Cas systems, as well as for adaptation of type I-E and I-F systems (19). Together, these results suggest that QS regulation of CRISPR-Cas may be a general phenomenon allowing bacteria to balance the risk of phage infection with the burden of producing CRISPR-Cas complexes and the risk of suicide from autoimmunity.

Here, using mutagenesis and molecular analyses, we show that another cue, low temperature, promotes CRISPR adaptation and interference in PA14. Specifically, at low temperature, CRISPR-Cas complex levels increase and growth rate decreases, each of which promotes increased adaptation. Using a QS mutant and synthetic QS compounds, we show that the temperature and QS inputs act synergistically. We hypothesize that the low temperature- and QS-mediated increases in CRISPR-Cas complex abundance elevate the number of possible adaptation/interference events. Furthermore, the reduced growth rate causes an apparent increase in CRISPR-Cas activity by providing the time required for the CRISPR-Cas machinery to carry out all of the required steps (adaptation, expression, and interference) for successful defense against a foreign invading element prior to cell division. If, simultaneously, the slow-growth conditions were unfavorable for phage propagation, it would give the CRISPR-Cas system the chance to accomplish the adaptation program prior to the cell becoming overwhelmed by replicating phage, making this mechanism particularly effective.

## RESULTS

### Temperature affects CRISPR adaptation.

P. aeruginosa is an environmental bacterium and an opportunistic human pathogen that causes nosocomial infections and chronic lung infections in patients with cystic fibrosis (CF) (20). PA14, the strain used in the present studies, was isolated from a burn victim. Additionally, PA14 is pathogenic in both plants and mice (21). PA14 has a type I-F CRISPR-Cas system, providing it with resistance to phage (22, 23).

Naive CRISPR adaptation to a phage that a bacterium has not encountered previously is rare in laboratory analyses of type I CRISPR-Cas systems (10). Adaptation requires cleavage of the foreign phage DNA, incorporation of a new spacer in the CRISPR array, transcription and processing into a mature crRNA, crRNA-guided detection of the foreign cDNA by the newly made crRNA in the Cas complex, and target cleavage by a Cas nuclease. Crucially, all of those steps must occur prior to the cell being overwhelmed by replicating phage DNA. We wondered if slowing bacterial growth could buy a bacterium more time for its CRISPR-Cas machinery to successfully adapt to and eliminate a foreign genetic element. This mechanism may be particularly relevant in the case of plasmids, which do not kill the host, and in the case of multiple phage infections of a single host cell, where the spacers acquired from one phage can be used against other invading phages. With this thought in mind, we were struck by the versatility of P. aeruginosa with respect to its ability to successfully reside both in the soil/plants and in mammalian hosts. These niches vary dramatically in many respects, notably for our present work, with regard to temperature and phage exposure (24, 25). We hypothesized that slow growth, as experienced by P. aeruginosa in the cooler temperatures of the phage-ridden soil or during plant infection, rather than the higher temperature experienced during human infection, could provide a favorable locale for CRISPR adaptation events to occur.

**FIG 1:**
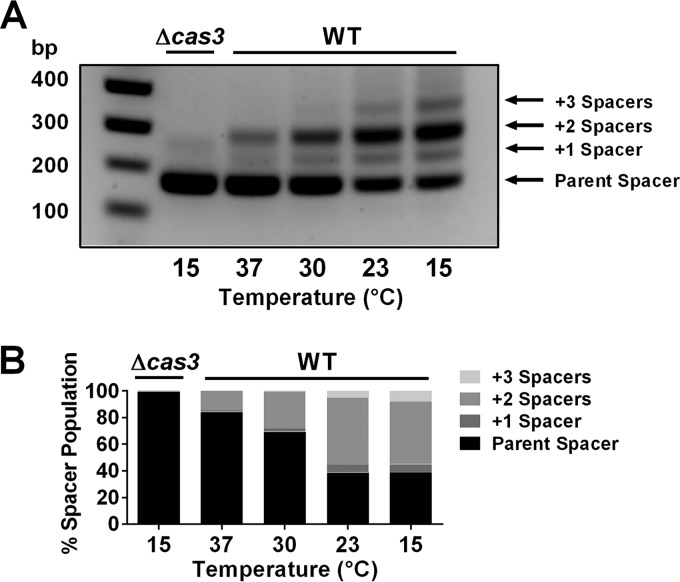
CRISPR adaptation is temperature dependent. Integration of new CRISPR spacers into the CRISPR2 locus was measured by PCR amplification of the CRISPR2 array region from single colonies of PA14 and the Δ*cas3* mutant. Both strains harbored the CRISPR-targeted plasmid, pCR2SP1 seed, containing a seed mutation that promotes adaptation. Each adaptation event results in acquisition of a new spacer (32 bp) and repeat (28 bp), which is exhibited by a 60-bp increase in size of the CRISPR locus and can be visualized by gel electrophoresis. (A) Adaptation of PA14 cells carrying pCR2SP1 seed at 37, 30, 23, or 15°C. The Δ*cas3* mutant is incapable of cleaving DNA bound by the Csy1–4 complex and serves as a negative control for adaptation. Data are shown for representative colonies. (B) Quantitation of the spacer population in panel A, *n* = 6.

To assess whether temperature influences CRISPR adaptation, we measured the ability of PA14 to adapt to the plasmid pCR2SP1 seed when grown at 37, 30, 23, and 15°C. The pCR2SP1 seed plasmid harbors a protospacer targeted by CRISPR2 spacer 1. The protospacer possesses a single base mutation in the 5′ seed region which fosters priming for CRISPR adaptation. During adaptation, new CRISPR spacers are incorporated at the 5′ end of a CRISPR array (1, 4). Thus, spacer number 1 becomes spacer number 2, and so on. This feature of the adaptation mechanism provides a convenient means to track adaptation events by PCR amplification of the expanding region. In our analysis, we used PCR primers flanking spacer number 1 of the CRISPR2 array. We separated the products based on size and visualized the CRISPR spacer population. In PA14, introduction of each new spacer and repeat adds 60 bp to the existing CRISPR array. In order to have minimal CRISPR-Cas activity at the start of the experiment, cultures of PA14 were grown at 37°C to a low cell density and transformed with pCR2SP1 seed (18). Transformants were allowed to recover at 37°C for 1 h and subsequently were grown on Luria-Bertani (LB) agar with gentamicin at the respective temperatures until they reached 1 mm in diameter. Single colonies were analyzed for adaptation events by PCR. Figure 1A shows that PA14 cells grown at 37°C have a spacer population consisting primarily of the unadapted parent array with a minor subpopulation that gained one or two new spacers. Cas3, which cleaves DNA when bound by the Csy1–4 complex, is required for adaptation to occur. Adaptation to the pCR2SP1 seed plasmid appears to favor the acquisition of two additional spacers rather than one or three additional spacers. Often, an adaptation ladder is observed in which each new/additional adaptation event is less frequent than the previous one; however, in the case of adaptation primed by plasmids, others have also shown adaptation patterns similar to those in Fig. 1A (11, 26). We sequenced the introduced spacers from 10 individual adaptation events and found that all new spacers were derived from the priming plasmid at locations within ∼1 kb of the protospacer (data not shown). Notably, for each decrease in growth temperature to 30, 23, and 15°C, PA14 contains a higher proportion of adapted arrays. Quantification of these adaptation events shows that approximately 16% of the spacer population is adapted when grown at 37°C, and this proportion increases to 61% for PA14 grown at 15°C (Fig. 1B). Moreover, the fraction of arrays that acquired multiple spacers also increases with decreasing growth temperature. Arrays containing three new spacers cannot be detected in cells grown at 37°C, whereas approximately 8% of the cells grown at 15°C have acquired three spacers.

We envision three possible mechanisms that could underlie the increase in CRISPR adaptation that occurs at low growth temperatures, as follows: (i) the CRISPR-targeted plasmid is present at higher copy number at low temperatures than at high temperatures, fostering higher rates of priming, and hence, an increase in potential adaptation events; (ii) CRISPR-Cas complexes are more abundant at low temperatures than at high temperatures, which again would increase the chances for adaptation; and (iii) the reduced bacterial growth rate at low temperatures compared to high temperatures could provide CRISPR-Cas complexes additional time to perform all of the steps necessary to achieve immunity and hence enable higher numbers of adaptation events to occur prior to each cell division. Any combination of these three mechanisms is also possible and could contribute to the connection between low temperature and high CRISPR adaptation frequency.

### Temperature does not affect pHERD30T copy number.

**FIG 2:**
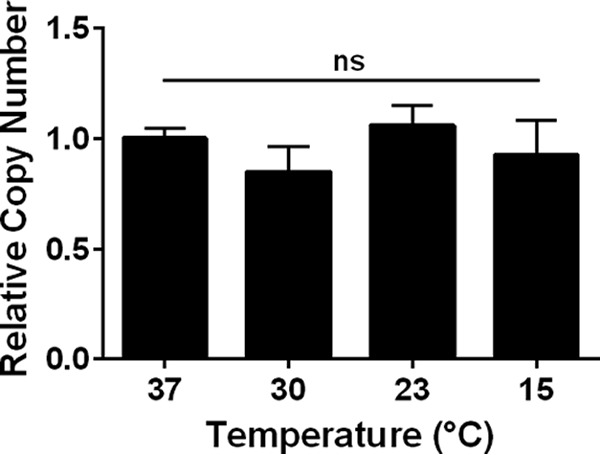
Temperature does not affect the relative copy number of pHERD30T in PA14. PA14 harboring pHERD30T, the empty vector backbone for the pCR2SP1 seed plasmid, was grown at 37, 30, 23, and 15°C on LB agar supplemented with gentamicin (50 μg/ml). The copy number of plasmid DNA relative to chromosomal DNA was measured by qPCR of total DNA using primers for pHERD30T and *rpoB*. Error bars represent standard deviation (SD) from *n* = 3 replicates (*P* = 0.1752, one-way analysis of variance [ANOVA]). ns, not significant.

First, we examined the possibility that temperature affects the relative copy number of the incoming plasmid. To do this, we measured, at different temperatures, the relative copy number of pHERD30T, the empty high-copy-number vector backbone for the pCR2SP1 seed plasmid (23). PA14 cells carrying pHERD30T were grown at 37, 30, 23, and 15°C. The relative copy number of pHERD30T was assayed by quantitative PCR (qPCR) of the total DNA using the chromosomal *rpoB* gene as the control. Figure 2 shows that there is no effect of temperature on the relative copy number of pHERD30T.

### Temperature has only a modest effect on Csy4 abundance.

We examined the second possibility, that the level of the CRIPSR-Cas machinery present in cells is affected by temperature. We reasoned that temperature could affect the transcription, translation, and/or stability of Cas proteins. No matter which mechanism, the outcome would be a change in the cellular concentration of CRISPR-Cas complexes. Thus, we assessed the relative abundances of CRISPR-Cas complexes at the different temperatures using Csy4-3×FLAG as the proxy. Csy4 is a component of the Csy1–4-crRNA CRISPR-Cas complex that binds to and targets foreign DNA for cleavage (22). A cross-streak assay was performed to verify that CRISPR-Cas-dependent resistance to the CRISPR-targeted phage DMS3m^vir^ was not affected by fusion of the 3×FLAG tag to Csy4 (see Fig. S1 in the supplemental material). In contrast to a ΔCRISPR Δ*cas* mutant strain lacking both CRISPR arrays, *cas1*, *cas3*, and *csy1*–*4* and which succumbed to DMS3m^vir^ phage infection, the PA14 strain carrying *csy4-3×flag* was resistant, suggesting that the Csy4-3×FLAG protein is functional.

10.1128/mBio.02184-18.1 FIG S1 The 3×FLAG-tagged Csy4 protein is functional. WT PA14, *csy4-3×flag* mutant, and ΔCRISPR Δ*cas* mutant strains were streaked (left to right) across the virulent phage DMS3m^vir^ (indicated by the vertical dotted line). DMS3m^vir^ is targeted by PA14 CRISPR2 spacer 1. A functional CRISPR-Cas apparatus protects PA14 cells against this phage (23). Download FIG S1, TIF file, 0.1 MB. Copyright © 2018 Høyland-Kroghsbo et al. 2018 Høyland-Kroghsbo et al. This content is distributed under the terms of the Creative Commons Attribution 4.0 International license.

**FIG 3:**
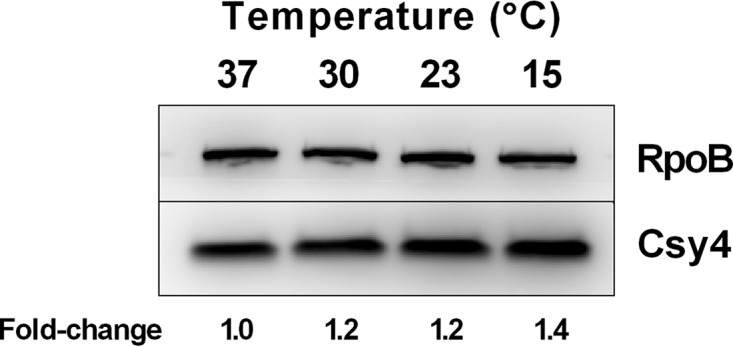
Csy4 levels are modestly upregulated at low temperatures. Western blot of PA14 Csy4-3×FLAG grown at 37, 30, 23, and 15°C. Top, abundance of RpoB, which was used as the endogenous control. Bottom, abundance of Csy4-3×FLAG. Quantitation of the relative abundance of Csy4-3×FLAG normalized to RpoB is shown below the blot. The data are representative of >3 independent experiments.

To test whether growth temperature affects Csy4 levels, we grew PA14 at 37, 30, 23, and 15°C and measured the relative abundance of Csy4 by Western blotting. Figure 3 shows that relative to a control protein, RpoB, Csy4-3×FLAG levels increase slightly with decreasing temperature. Quantitation shows that there is 1.2- to 1.4-fold more Csy4-3×FLAG present at temperatures below 37°C (Fig. 3). To address the possibility that temperature affects Csy4-3×FLAG stability, in our assay, we grew WT PA14 carrying Csy4-3×FLAG at 23°C to an optical density at 600 nm (OD_600_) of 1. We treated the culture with gentamicin to arrest protein synthesis, divided the culture into two aliquots, and incubated one aliquot at 23°C and the other at 37°C for two more hours. Csy4 levels remained constant at both temperatures (Fig. S2). Using this assay, we cannot, however, exclude the possibility that there may be temperature-dependent differences in the stability of CRISPR-Cas complexes, which could contribute to the temperature-dependent regulation of CRISPR-mediated adaptation. We suggest that the enhanced CRIPSR-mediated adaptation that occurs at low temperatures (Fig. 1) can be explained by differences in the abundance of CRISPR-Cas machinery alone or, perhaps, in combination with slow growth, as investigated in the next section.

10.1128/mBio.02184-18.2 FIG S2 Csy4-3×FLAG stability at 37°C and 23°C. Western blot of PA14 Csy4-3×FLAG. Cultures were grown to an OD_600_ of 1 at 23°C, gentamicin was added to arrest protein synthesis, and the cultures were divided into two aliquots, where one was incubated at 37°C and the other at 23°C. Samples were taken at 0, 30, 60, and 120 min after the addition of gentamicin. Top, abundance of RpoB, which was used as the endogenous control. Bottom, abundance of Csy4-3×FLAG. Download FIG S2, TIF file, 0.1 MB. Copyright © 2018 Høyland-Kroghsbo et al. 2018 Høyland-Kroghsbo et al. This content is distributed under the terms of the Creative Commons Attribution 4.0 International license.

### Growth rate affects CRISPR-Cas activity.

We examined the final possibility, that growth rate contributes to the temperature-dependent effects we observe on CRISPR adaptation in PA14. For this analysis, we assayed the elimination of a plasmid rather than a phage because temperature affects CRISPR-Cas-independent phage-host interactions, including phage adsorption rates, plaquing efficiency, the lysis-versus-lysogeny switch, and the activity of restriction modification antiphage defenses (23, 27–30). Indeed, as one example of these complexities, we show in Fig. S3 that the rate of JBD44a phage adsorption to PA14 is increased at 23°C compared to 37°C, irrespective of the presence/absence of CRISPR-Cas. This temperature-mediated effect on adsorption may be explained by increased long-chain O-antigen decoration of the cell surface that occurs at low temperatures. These O-antigen moieties play phage receptor roles (31, 32).

10.1128/mBio.02184-18.3 FIG S3 Temperature affects phage adsorption independently of CRISPR-Cas. WT PA14, a ΔCRISPR Δ*cas* mutant, and a WT resistant (WT^r^) strain carrying a CRISPR spacer targeting JBD44a were grown to an OD_600_ of 1 at 37°C (A) or 23°C (B) and infected with phage JBD44a at a multiplicity of infection (MOI) of 30. PFU were quantified over time. SD represents 3 replicates. Cells grown at 23°C adsorb phage at a higher rate than those grown at 37°C. Download FIG S3, TIF file, 0.01 MB. Copyright © 2018 Høyland-Kroghsbo et al. 2018 Høyland-Kroghsbo et al. This content is distributed under the terms of the Creative Commons Attribution 4.0 International license.

To measure the influence of growth rate on the ability of CRISPR-Cas to eradicate a foreign genetic element, we assayed CRISPR-Cas effectiveness in eliminating the CRISPR-targeted plasmid called pCR2SP1 (23) when PA14 was grown at different rates. This pHERD30T-derived plasmid contains a protospacer targeted by CRISPR2 spacer 1 flanked by a PAM sequence that is required for CRISPR interference (7).

**FIG 4:**
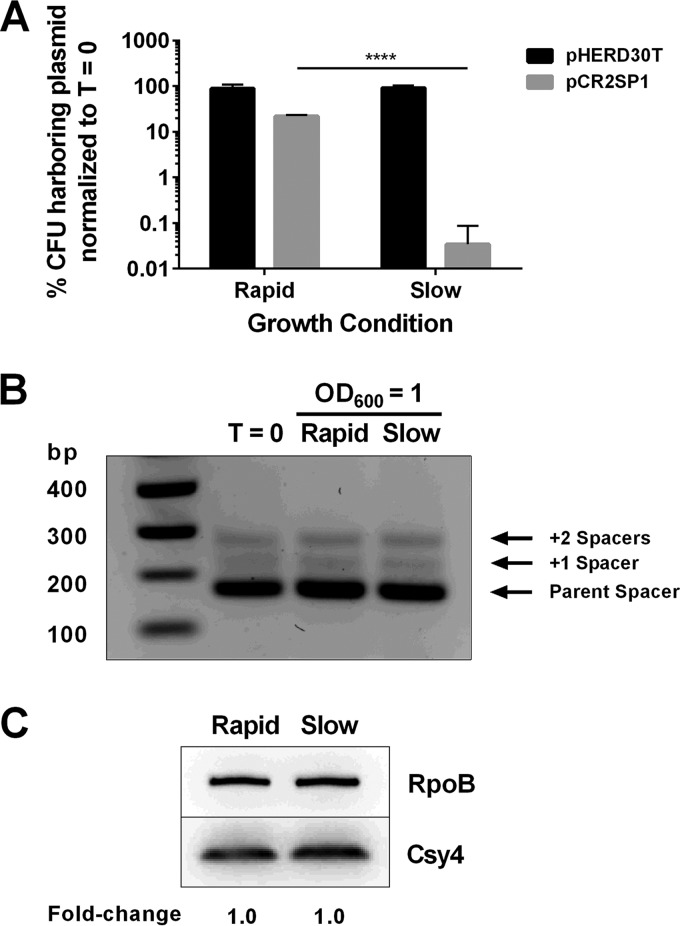
Growth rate affects CRISPR-Cas activity. (A) Retention of the control parent plasmid pHERD30T (black) and the CRISPR-targeted plasmid pCR2SP1 (gray) in PA14 grown at 37°C to an OD_600_ of 1 with aeration at 250 rpm (denoted rapid growth) or 150 rpm (denoted slow growth). One hundred percent denotes no plasmid loss. SD represents 3 replicates. (*P* < 0.0001, Student’s *t* test). (B) Adaptation of PA14 cells carrying pCR2SP1 grown as in panel A at T = 0 and at the end of the experiment, at OD_600_ of 1. (C) Western blot of PA14 Csy4-3×FLAG grown as in panel A to an OD_600_ of 1. Top, abundance of RpoB, which was used as the endogenous control. Bottom, abundance of Csy4-3×FLAG. Quantitation of the relative abundance of Csy4-3×FLAG normalized to RpoB is shown below the blot.

To control bacterial growth rate, we varied aeration levels by shaking the cultures at either 250 rpm or at 150 rpm. We note that we did not use minimal versus rich growth medium to control growth rate because, as noted above, nutrient availability affects the activity of CRISPR-Cas systems in multiple bacteria, including PA14 (14–17, 33). Rather, we grew the rapidly/slowly shaken samples for different times to enable the cultures to achieve the same final cell density (Fig. S4). We quantified the percentages of the control plasmid pHERD30T and the CRISPR-targeted plasmid pCR2SP1 present in the cells at the beginning of the experiment and assessed their retention after rapid or slow growth at 37°C to an OD_600_ of 1. In spite of CRISPR targeting of pCR2SP1, after rapid growth, 22% of the plasmids were retained compared to time zero. In contrast, following slow growth, only 0.03% of the plasmids were retained. Thus, CRISPR-Cas interference was >600-fold more effective during slow growth than during rapid growth (Fig. 4A). Upon extended growth of the rapidly growing culture, the effectiveness of CRISPR-Cas interference increased to levels similar to those of the slowly growing culture (Fig. S5). The pCR2SP1 plasmid carries a protospacer with perfect CRISPR targeting; however, it may also prime adaptation, which in turn would further increase the frequency of CRISPR targeting and subsequent plasmid curing. In order to address the possibility of priming from the protospacer contributing to the plasmid curing measured in Fig. 4A, we quantified the population-wide level of adaptation at both the beginning and the end of the experiment. Figure 4B shows that no increase in adaptation frequency occurred during the experiment. Hence, the plasmid curing observed in Fig. 4A can be attributed to CRISPR targeting of the pCR2SP1 plasmid. To determine whether growth rate affects Csy4 levels, we assessed Csy4-3×FLAG amounts following growth at 37° with shaking at 150 rpm and 250 rpm to an OD_600_ of 1. Figure 4C shows that growth rate does not affect the abundance of Csy4-3×FLAG.

10.1128/mBio.02184-18.4 FIG S4 Growth curves for PA14 grown at 150 rpm (slow) and 250 rpm (rapid). Colonies of PA14 carrying the pHERD30T plasmid were inoculated in LB broth and grown at 37°C with rapid (250 rpm) or slow (150 rpm) shaking. Growth was measured as the OD_600_. The doubling time of PA14 during rapid exponential growth is 32.6 min (*R*^2^ = 0.9996). During slow exponential growth, between an OD_600_ of 0.025 and 0.2, the doubling time is 61.4 min (*R*^2^ = 0.9791), and between OD_600_ of 0.2 and 0.7, the doubling time is 185.3 min (*R*^2^ = 0.9546). *n* = 3. Download FIG S4, TIF file, 0.01 MB. Copyright © 2018 Høyland-Kroghsbo et al. 2018 Høyland-Kroghsbo et al. This content is distributed under the terms of the Creative Commons Attribution 4.0 International license.

10.1128/mBio.02184-18.5 FIG S5 Plasmid loss after extended growth. The data in this figure extend the analyses shown in Fig. 4A. In Fig. 4A, cultures of PA14 carrying pCR2SP1 were grown at 37°C with shaking at 150 rpm (slow growth) or 250 rpm (rapid growth) until each culture reached an OD_600_ of 1. Here, we show data for cultures grown at 250 rpm for an extended time, a time equal to that required for the cultures grown at 150 rpm to reach OD_600_ of 1. The data show that increased growth time at 250 rpm, which enables the culture to enter stationary phase, causes increased loss of both the untargeted pHERD30T plasmid and the CRISPR-targeted pCR2SP1 plasmid. Download FIG S5, TIF file, 0.05 MB. Copyright © 2018 Høyland-Kroghsbo et al. 2018 Høyland-Kroghsbo et al. This content is distributed under the terms of the Creative Commons Attribution 4.0 International license.

### Quorum sensing acts synergistically with temperature to regulate CRISPR-Cas-mediated adaptation.

QS regulates CRISPR-Cas (18, 19). QS relies on the production, release, and group-wide detection of diffusible signaling molecules called autoinducers (AIs), and the process allows bacteria to collectively control genes required for group behaviors (34). The major PA14 QS circuit consists of two AI-receptor pairs called LasI/R and RhlI/R. LasI produces the AI 3-oxo-C_12_-homoserine lactone (3OC_12_-HSL), which activates the receptor LasR. LasR activates expression of many genes, including those encoding the second QS system, *rhlI* and *rhlR* (35–38). RhlI synthesizes the AI C_4_-homoserine lactone (C_4_-HSL) that, when bound to RhlR, activates a second wave of QS genes (36).

**FIG 5:**
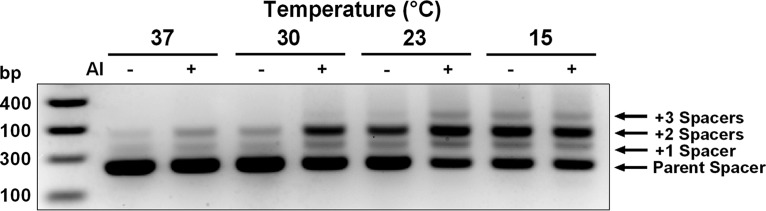
QS and low temperature act synergistically to enhance CRISPR-Cas-mediated adaptation. PCR amplification of the CRISPR2 array and visualization by gel electrophoresis, as in Fig. 1. The PA14 Δ*lasI* Δ*rhlI* mutant does not produce 3OC_12_-HSL or C_4_-HSL. 3OC_12_-HSL and C_4_-HSL (designated AI) were supplied at saturating concentrations (2 μM and 10 μM, respectively), as denoted. The data are representative of >3 independent experiments.

We previously showed that in PA14, *cas3* expression is activated at high cell density, a hallmark of a QS-regulated trait. Moreover, a PA14 mutant lacking both QS AI synthase genes (Δ*lasI* and Δ*rhlI*) exhibited reduced CRISPR-Cas expression, interference, and adaptation compared to WT PA14. Complementation of the Δ*lasI* Δ*rhlI* mutant via exogenous supplementation with the 3OC_12_-HSL and C_4_-HSL AIs restored CRISPR-Cas function to WT levels (18). We wondered whether the temperature and QS cues function independently or synergistically to regulate CRISPR-Cas. To assess if QS influences temperature-dependent CRISPR adaptation, we used an adaptation assay similar to that shown in Fig. 1. In this case, we assayed CRISPR-Cas-mediated adaptation to the pCR2SP1 seed plasmid in a PA14 Δ*lasI* Δ*rhlI* mutant that lacks the ability to produce the two QS AIs. Colonies were grown in the absence and presence of 2 μM 3OC_12_-HSL plus 10 μM C_4_-HSL. Compared to the untreated Δ*lasI* Δ*rhlI* mutant strain, supplementation with AIs increased the frequency of CRISPR adaptation (Fig. 5). The effect of AIs on adaptation frequency was more pronounced at 30°C and 23°C than at 37°C, suggesting that QS and lower temperatures synergistically enhance CRISPR-Cas-mediated adaptation. At 15°C, however, there was no effect of AI supplementation on CRISPR-Cas-directed adaptation frequency, suggesting that at the lowest temperature we tested, temperature alone is sufficient to promote the maximum operating capacity of the CRISPR-Cas system. To examine the role of QS in the temperature-dependent production of Csy4, we measured Csy4-3×FLAG levels in the WT and in the Δ*lasI* Δ*rhlI* mutant strain grown at 37, 30, 23, and 15°C. Because RpoB production is altered in the Δ*lasI* Δ*rhlI* mutant strain relative to WT PA14, we used GroEL as the internal control for this experiment. Figure S6A shows that at all temperatures tested, levels of Csy4-3×FLAG are higher in the WT strain than in the Δ*lasI* Δ*rhlI* mutant strain. Additionally, reducing the temperature promotes increases in Csy4-3×FLAG levels, and this effect is more pronounced in the WT strain than in the Δ*lasI* Δ*rhlI* mutant strain. In agreement with the QS and temperature-dependent findings regarding the regulation of Csy4-3×FLAG, interference is more effective in the WT strain than in the Δ*lasI* Δ*rhlI* mutant strain when grown rapidly or slowly (Fig. S6B, right).

10.1128/mBio.02184-18.6 FIG S6 The effect of QS on temperature-dependent Csy4 regulation and growth rate regulation of CRISPR-Cas activity. (A) Western blot of PA14 Csy4-3×FLAG grown at 37, 30, 23, and 15°C. Top, abundance of GroEL, which was used as the endogenous control. Bottom, abundance of Csy4-3×FLAG. Quantitation of the relative abundance of Csy4-3×FLAG normalized to GroEL is shown below the blot. (B) Retention of the control parent plasmid pHERD30T (left) and the CRISPR-targeted plasmid pCR2SP1 (right) in WT PA14 and the Δ*lasI* Δ*rhlI* mutant strain grown at 37°C to an OD_600_ of 1 with aeration at 250 rpm (denoted rapid growth) or 150 rpm (denoted slow growth). One hundred percent denotes no plasmid loss. SD represents 3 replicates. (*P* < 0.001 for pCR2SP1, Student’s *t* test). Download FIG S6, TIF file, 0.2 MB. Copyright © 2018 Høyland-Kroghsbo et al. 2018 Høyland-Kroghsbo et al. This content is distributed under the terms of the Creative Commons Attribution 4.0 International license.

Li et al. reported that in PA14, CRISPR-Cas degrades *lasR* mRNA, which in turn affects QS-controlled virulence in a mouse model of infection (39). Therefore, the possibility existed that temperature control of CRISPR-Cas activity could affect LasR receptor levels, resulting in a regulatory feedback loop that contributes to the effects we observe. We could not, however, replicate the findings by Li et al. Specifically, reverse transcription-quantitative PCR (qRT-PCR) of *lasR* in the PA14 WT and ΔCRISPR Δ*cas* mutant strains showed no differences under any of our experimental conditions (Fig. S7A). We also tested the relative levels of *lasB*, encoding elastase, which is under the direct control of LasR (Fig. S7B). Again, there was no effect of CRISPR-Cas on expression of this QS-regulated gene under any condition we tested.

10.1128/mBio.02184-18.7 FIG S7 CRISPR-Cas does not regulate *lasR* or *lasB*. (A) Relative *lasR* mRNA levels normalized to 5S RNA were measured by qRT-PCR in PA14 and in the isogenic ΔCRISPR Δ*cas* mutant strain under different QS states (OD_600_, 1 and 2). (B) As in panel A, showing *lasB* mRNA, encoding the virulence factor elastase, the transcription of which is activated by LasR. Error bars represent SD from *n* = 3 replicates. (*P* > 0.05, Student’s *t* test). Unlike the results obtained by Li et al. (39), under our experimental conditions, CRISPR-Cas neither affects the relative levels of *lasR* mRNA nor LasR-regulated *lasB* mRNA levels. Download FIG S7, TIF file, 0.02 MB. Copyright © 2018 Høyland-Kroghsbo et al. 2018 Høyland-Kroghsbo et al. This content is distributed under the terms of the Creative Commons Attribution 4.0 International license.

## DISCUSSION

Here, we discover that temperature is an environmental regulator of CRISPR-Cas interference and adaptation, i.e., target cleavage and acquisition of new immunity spacers, respectively, in P. aeruginosa PA14. Specifically, CRISPR adaptation increases with decreasing temperature. The underlying mechanism appears to rely minimally on an increased abundance of CRISPR-Cas components, but rather primarily on slow growth itself, presumably buying time for the CRISPR-Cas machinery to successfully destroy/adapt to foreign DNA. Given that growth rate and temperature are often connected, this phenomenon could be relevant in other bacteria that harbor CRISPR-Cas systems.

P. aeruginosa is a soil-dwelling organism that is also an opportunistic human pathogen. Phage abundance and diversity are high in ecosystems, such as soil, whereas they are particularly limited in the human lung, a major habitat for infectious P. aeruginosa, most notably in CF sufferers (24, 25). Our finding that higher CRISPR-Cas activity occurs at lower temperatures correlates with the increased threat of potential foreign parasitic elements in soil (generally at low temperatures) compared to during human infection (generally at 37°C). Thus, we predict that bacteria are better equipped to defend themselves against phage attacks and to adapt to mutating phage in the environment than in the human body; however, one consequence is that bacteria may be vulnerable to a higher incidence of autoimmunity in the environment. We recognize that P. aeruginosa causes biofilm infections, in which the bacteria exhibit slow growth (40), and that not all P. aeruginosa infections in humans are under 37°C conditions. We anticipate that CRISPR-Cas may be more active in P. aeruginosa biofilm infections and in superficial wound infections than during bacteremia, which would presumably involve planktonic cells at 37°C. Our discovery that low temperatures increase CRISPR adaptation in P. aeruginosa suggests that the rarely occurring but crucial naive adaptation events may take place predominantly during the bacterium’s soil dwelling existence and during slow growth in biofilms.

Another environmental regulator of CRISPR-Cas in P. aeruginosa PA14 is QS. We previously showed that QS, via the two main synthases LasI and RhlI, activates CRISPR-Cas expression, interference, and adaptation (18). Interestingly, in P. aeruginosa PAO1, QS is temperature dependent due to the presence of conserved thermosensing RNA “thermometers” that reside in the 5′ untranslated regions (UTRs) of *lasI* and *rhlAB-R*, the latter of which controls the expression of *rhlR*. Thermoregulation of *lasI* and *rhlR* increases their expression at 37°C compared to 30°C, and consequently, causes increased production of QS-regulated virulence factors (41). Given that, in PAO1, QS regulators and QS-controlled traits are more highly expressed at 37°C than at lower temperatures, one would expect CRISPR-Cas activity to be maximal at 37°C, since we found that QS activates CRISPR-Cas (18). Nonetheless, we do not find this to be the case, at least for PA14. CRISPR-Cas is more active at low temperatures than at high temperatures, and Csy4 is 20 to 40% more abundant at low temperatures than at high temperatures. To reconcile these findings, we posit that although the Csy4-3×FLAG protein displays equal stability for up to 2 h at 37°C and 23°C, CRISPR-Cas complexes may be more stable at lower temperatures than at higher temperatures, which yields increased levels of assembled CRISPR-Cas complexes, and thus, CRISPR-Cas activity, at low temperatures. Additionally, low temperatures may favor more rapid and tighter annealing of crRNA to its target and thereby enhance interference and adaptation.

Other reports show correlations between slow growth and CRISPR adaptation. For example, in nutrient-poor medium, PA14 primarily acquires CRISPR-based immunity to the phage DMS3^vir^ as opposed to during growth in nutrient-rich medium, which, in contrast, promotes the accumulation of phage receptor mutations (17). We hypothesize that, under poor growth conditions, increased CRISPR-based adaptation is mediated in part by the lower growth rate of the bacteria than that under ideal growth conditions, again providing the bacteria crucial time to adapt. In the same vein, Amlinger et al. discovered that naive adaptation occurs with the highest frequency in late-exponential phase in liquid cultures of Escherichia coli overproducing CRISPR-Cas (42). One can imagine that CRISPR-*cas* expression was maintained at a relatively constant level in this experiment due to the use of a synthetic promoter. Thus, maximal adaptation in late-exponential phase could be due to the declining growth rate, in agreement with our findings (Fig. 4).

Our results showing that slow growth increases the frequency of CRISPR-Cas-mediated plasmid loss suggests that biofilms, which exhibit particularly slow growth in their cores (40, 43), may exhibit exceptionally high CRISPR-Cas activity compared to exponentially growing planktonic cells. High CRISPR-Cas activity in slowly growing cells offers the exciting possibility that transiently growth-arrested antibiotic-tolerant persister subpopulations (44) could be natural reservoirs of cells that are primed for CRISPR adaptation. A phage, upon infecting a persister cell, has its lytic cycle arrested until the host cell resumes growth (45). If CRISPR-Cas is active in persister cells, the persistent stage would afford the host cell ample time for adaptation, possibly providing the cell a means to more efficiently eliminate additional phages that have also infected the cell. Moreover, after resuming growth, the adapted cell would be prepared to fend off infecting phages coming from neighboring cells. Indeed, persistence, which is induced at high cell density when cells are at highest risk of phage infection, may be a form of phage defense. QS increases persister formation in PA14 and PAO1 (46). Thus, QS inhibitors may reduce the fraction of persister cells present, which would (i) allow antibiotics to kill pathogenic bacteria more effectively and (ii) minimize the population of cells that could be particularly prone to acquiring CRISPR-based immunity toward phage therapies. We envision that antibacterial treatments consisting of QS inhibitors, antibiotics, and phage therapies could exhibit exceptional synergy in killing pathogens, including the notorious persister cells. Last, and in contrast, our findings offer simple conditions, namely decreasing growth rate and/or reducing temperature, that could be explored to increase CRISPR adaptation frequency for applications, such as the development of phage-resistant bacterial strains, possibly for use in industry or as probiotics.

## MATERIALS AND METHODS

### Bacterial strains, plasmids, and phage.

The strains and plasmids used in this study are listed in Table S1. To construct the chromosomal 3*×flag*-tagged *csy4* in PA14, DNA fragments flanking the 3′ terminus of *csy4,* including a 3*×flag* tag, were amplified, sewed together by overlap extension PCR, and cloned into pEXG2 (a generous gift from Joseph Mougous, University of Washington, Seattle, WA) using HindIII and XbaI restriction sites (47). The plasmid to make the ΔCRISPR Δ*cas* PA14 mutant was engineered using the identical strategy and DNA fragments surrounding the CRISPR *cas* locus, flanked by EcoRI and XbaI restriction sites. The resulting plasmids were used to transform E. coli SM10λ*pir* and were subsequently mobilized into PA14 via mating. Exconjugants were selected on Luria-Bertani (LB) containing gentamicin (30 μg/ml) and irgasan (100 μg/ml), followed by recovery of mutants on M9 medium containing 5% (wt/vol) sucrose. Candidate mutants were confirmed by PCR and sequencing. A PA14 strain harboring a CRISPR spacer matching phage JBD44a was generated by cloning 1,581 bp of JBD44a gene *gp*33 using native HindIII sites into the adaptation-promoting pCR2SP1 seed plasmid (18). The plasmid was propagated in PA14 on LB agar with 50 µg/ml gentamicin, and single colonies were streaked repeatedly on LB agar and tested for plasmid loss. CRISPR-adapted clones were identified using PCR with primers that enabled an assessment of incorporation of spacers into either CRISPR1 or CRISPR2. Newly integrated spacers were identified and mapped using sequencing. A strain with the new spacer AGCCACAACANAGGCCAGAGAAGCTGCTGCGA in CRISPR2 that targeted gene *gp33* was selected and tested for resistance to JBD44a by a cross-streak assay. The primers used are listed in Table S2.

10.1128/mBio.02184-18.8 TABLE S1 Bacterial strains, phage, and plasmids. Download Table S1, PDF file, 0.05 MB. Copyright © 2018 Høyland-Kroghsbo et al. 2018 Høyland-Kroghsbo et al. This content is distributed under the terms of the Creative Commons Attribution 4.0 International license.

10.1128/mBio.02184-18.9 TABLE S2 Primers used in this study. Download Table S2, PDF file, 0.04 MB. Copyright © 2018 Høyland-Kroghsbo et al. 2018 Høyland-Kroghsbo et al. This content is distributed under the terms of the Creative Commons Attribution 4.0 International license.

### Growth conditions.

PA14 and mutants were grown at the indicated temperatures in LB broth or on LB solidified with 15 g agar/liter. LB was supplemented with 50 μg/ml gentamicin where appropriate. For AI supplementation assays, 3OC_12_-HSL and C_4_-HSL (Sigma) or the solvent dimethyl sulfoxide (DMSO) was used. Growth of bacterial cultures was measured by OD_600_, where 1 unit equals 10^9^ CFU/ml.

### Adaptation assay.

WT PA14 and the Δ*lasI* Δ*rhlI* mutant were transformed with pCR2SP1 seed, as described previously (18), and plated on LB medium containing 50 μg/ml gentamicin; in the case of the Δ*lasI* Δ*rhlI* mutant, either DMSO (control) or 2 μM 3OC_12_-HSL plus 10 μM C_4_-HSL (designated AI) was added. The plates were incubated at 37, 30, 23, and 15°C until the colonies were 1 mm in diameter. Single colonies were tested for population-wide integration of new CRISPR spacers by PCR using DreamTaq Green PCR master mix (Thermo Fisher); primers used were designed to anneal upstream of the CRISPR2 array and inside the second spacer, which enabled the detection of expansion of this array. The PCR products were subjected to agarose gel electrophoresis, and band intensities were analyzed using the ImageQuant TL software (GE Healthcare).

### Relative plasmid copy number.

PA14 harboring pHERD30T, the empty vector backbone for the pCR2SP1 seed plasmid, was grown at 37, 30, 23, and 15°C on LB agar supplemented with gentamicin (50 μg/ml). Total DNA was extracted from individual colonies that were 1 mm in size using a DNeasy blood and tissue kit (Qiagen). qPCR was performed using PerfeCTa SYBR Green FastMix, Low ROX (Quantabio) with primers specific for pHERD30T and chromosomal *rpoB*.

### Western blot analysis.

The PA14 *csy4*-3*×flag* and Δ*lasI* Δ*rhlI csy4*-*3×flag* mutant strains were streaked onto LB medium and grown at 37, 30, 23, or 15°C until individual colonies reached 1 mm in diameter. Single colonies were harvested and lysed with BugBuster protein extraction reagent (Millipore), following the manufacturer’s instructions. Fifty micrograms of protein was separated by SDS-PAGE on a 4 to 20% Mini-PROTEAN TGX polyacrylamide gel (Bio-Rad) and blotted onto a polyvinylidene difluoride (PVDF) membrane (catalog no. 1620174; Bio-Rad). The membrane was incubated for 1 h with monoclonal Anti-FLAG M2-peroxidase (HRP) antibody (catalog no. A8592; Sigma), monoclonal anti-RpoB antibody (catalog no. ab191598; Abcam), both at a 1:3,000 dilution, or polyclonal anti-GroEL antibody (catalog no. G6532; Sigma) at a 1:15,000 dilution in Tris-buffered saline with Tween 20 (TBST) and 5% skim milk. Anti-rabbit antibody (catalog no. W4011; Promega) was used as the secondary antibody for detection of the anti-RpoB and anti-GroEL antibodies. The membrane was washed in TBST and was developed using SuperSignal West Femto maximum sensitivity substrate (catalog no. 34095; Thermo Scientific).
