# Supplementary material for: Temperature, by Controlling Growth Rate, Regulates CRISPR-Cas Activity in Pseudomonas aeruginosa
Source: mBio. 2018 Nov 13;9(6):e02184-18. doi: 10.1128/mBio.02184-18 (PMC6234860; doi:10.1128/mBio.02184-18)
Supplement: TABLE S1 [file mbo006184172st1.pdf]

Supplementary Table S1. Bacterial strains, phage, and plasmids.

Strain, phage, or plasmid	Description	Source
UCBPP-PA14	Generous gift from George O'Toole, Geisel School of Medicine at Dartmouth University, Hanover, NH	Laboratory stock
NMHK215	PA14 WT^R, PA14 carrying a CRISPR2 spacer targeting JBD44a	This study
NMHK326	PA14 ΔCRISPR Δ*cas*	This study
NMHK383	PA14 *csy4-3xflag*	This study
NMHK396	PA14 Δ*lasI* Δ*rhlI csy4-3xflag*	This study
SM53	PA14 Δ*lasI* Δ*rhlI*	(1)
SMC4268	PA14 Δ*cas3*	(2)
E. coli SM10λ*pir*	*thi thr leu tonA lacY supE recA*::RP4-2-Tc::Mu	Laboratory stock
pEXG2	Allelic exchange vector with pBR origin, gentamicin resistance, *sacB*, Generous gift from Joseph Mougous, University of Washington, Seattle	(3)
JBD44a	Generous gift from Joseph Bondy-Denomy, University of California, San Francisco	(4)
$DMS3m^{vir}$	Phage targeted by CRISPR2 spacer 1 (virulent mutant of $DMS3^{100\%}$)	(5)
pHERD30T	Empty plasmid, gentamicin resistance	(5)

pCR2SP1	pHERD30T containing the protospacer to CRISPR2 spacer 1	(5)
pCR2SP1 seed	pCR2SP1, with a one base seed mutation	(1)

Supplemental references for Supplementary Table S1

1. Hoyland-Kroghsbo NM, Paczkowski J, Mukherjee S, Broniewski J, Westra E, Bondy-Denomy J, Bassler BL. 2017. Quorum sensing controls the Pseudomonas aeruginosa CRISPR-Cas adaptive immune system. Proc Natl Acad Sci U S A 114:131-135.
2. Cady KC, O'Toole GA. 2011. Non-identity-mediated CRISPR-bacteriophage interaction mediated via the Csy and Cas3 proteins. J Bacteriol 193:3433-45.
3. Rietsch A, Vallet-Gely I, Dove SL, Mekalanos JJ. 2005. ExsE, a secreted regulator of type III secretion genes in Pseudomonas aeruginosa. Proc Natl Acad Sci U S A 102:8006-11.
4. Phee A, Bondy-Denomy J, Kishen A, Basrani B, Azarpazhooh A, Maxwell K. 2013. Efficacy of bacteriophage treatment on Pseudomonas aeruginosa biofilms. J Endod 39:364-9.
5. Cady KC, Bondy-Denomy J, Heussler GE, Davidson AR, O'Toole GA. 2012. The CRISPR/Cas adaptive immune system of Pseudomonas aeruginosa mediates resistance to naturally occurring and engineered phages. J Bacteriol 194:5728-38.
