# Supplementary material for: Temperature, by Controlling Growth Rate, Regulates CRISPR-Cas Activity in Pseudomonas aeruginosa
Source: mBio. 2018 Nov 13;9(6):e02184-18. doi: 10.1128/mBio.02184-18 (PMC6234860; doi:10.1128/mBio.02184-18)
Supplement: TABLE S2 [file mbo006184172st2.pdf]

Supplementary Table S2. Primers used in this study.

Primer	Sequence 5'-3'	Source
csy4 3x*flag* BamHI F	TCAGGATCCCCGTATCAACCATATCCGCC	This study
csy4 3x*flag* R	CTTTATAATCACCGTCATGGTCTTTGTAGTCGCTGCCGGAGCCGCTG CCGGAGCCGAACCAGGGAACGAAACCTC	This study
csy4 3x*flag* F	CCATGACGGTGATTATAAAGATCATGACATCGATTACAAGGATGAC GATGACAAGTGAAGAACCCCGTCAGCGCC	This study
csy4 3x*flag* XbaI R	GTCTCTAGAAGATACGTGCACAGATCGGA	This study
csy4 3x*flag* check F	ACAGCACTTCCGTCTCTTCA	This study
csy4 3x*flag* check R	CCTTTCCATGGGACTTCGTT	This study
CRISPR-*cas* EcoRI F	CCCGAATTCCTACACACCCCGAAAACACC	This study
CRISPR-*cas* up R	AACTTGCTGATTTATAAGGGCGTAAGAAATCGTCCGAAAAAAGGGT CGG	This study
CRISPR-*cas* down F	CGCCCTTATAAATCAGCAAGTTTAGACCAAAACCCCATCAACCTT	This study
CRISPR-*cas* XbaI R	CTAGTCTAGAAAATTCACCCAGAGCTTGCC	This study
CRISPR-*cas* check F	CGATGAGAAAGAAGAGCCCG	This study
CRISPR-*cas* check R	ATGGAGAACACAGGGGATGG	This study
gp33 JBD44 F	CTGAGAATGTCGGCAGCATC	This study
gp33 JBD44 R	CCGCGATCTCCTCTTTTGTG	This study
CRISPR1 adapt F	TTGGGGCTTGGAAGGTTGAT	(1)

CRISPR1 adapt R	AAGGCCAGCGCGCCGGTGAT	(1)
CRISPR2 adapt F	GAGGGTTTCTGGCGGGAA	(1)
CRISPR2 adapt R	GTCCAGAAGTCACCACCCG	(1)
pHERD30T qPCR F	TGCAAGGCGATTAAGTTGGG	This study
HERD30T qPCR R	CCTCTAGAGTCGACCTGCAG	This study
rpoB qPCR F	ATCATTCCTTACCGCGGTTC	This study
rpoB qPCR R	GAGGATCTCTTCGGTGCTGT	This study
lasR qPCR F	ACAGCCAGGACTACGAGAAC	This study
lasR qPCR R	CCCAGAAAATCGGCAGTACG	This study
lasB qPCR F	AGACCGAGAATGACAAAGTGGAA	(2)
lasB qPCR R	GGTAGGAGACGTTGTAGACCAGTTG	(2)
5S qPCR F	GAACCACCTGATCCCTTCCC	This study
5S qPCR R	TAGGAGCTTGACGATGACCT	This study

Supplemental references for Supplementary Table S2

1. Hoyland-Kroghsbo NM, Paczkowski J, Mukherjee S, Broniewski J, Westra E, Bondy-Denomy J, Bassler BL. 2017. Quorum sensing controls the Pseudomonas aeruginosa CRISPR-Cas adaptive immune system. Proc Natl Acad Sci U S A 114:131-135.
2. Crabbe A, De Boever P, Van Houdt R, Moors H, Mergeay M, Cornelis P. 2008. Use of the rotating wall vessel technology to study the effect of shear stress on growth behaviour of Pseudomonas aeruginosa PA01. Environmental Microbiology 10:2098-2110.
